# The Effect of Thermal-Softened Endotracheal Tubes on Postoperative Sore Throat and Other Complications—A Systematic Review and Meta-Analysis

**DOI:** 10.3390/jcm14113620

**Published:** 2025-05-22

**Authors:** Hui-Zen Hee, Chen-Hsi Chiu, Cheng-Wei Lu

**Affiliations:** 1 Department of Anesthesiology, Far Eastern Memorial Hospital, Banqiao District, New Taipei City 220, Taiwan; femh98940@femh.org.tw (H.-Z.H.); femh96629@femh.org.tw (C.-H.C.); 2 Department of Mechanical Engineering, Yuan Ze University, Taoyuan 320, Taiwan

**Keywords:** equipment design, hoarseness, intubation, intratracheal, laryngeal diseases, pharyngitis, postoperative complications

## Abstract

**Background**: Endotracheal tube (ETT) intubation during general anesthesia (GA) is commonly associated with postoperative sore throat. This study aimed to evaluate whether thermal-softened ETTs reduce the postoperative sore throat incidence in patients undergoing elective surgery under GA. **Methods**: We conducted a comprehensive search of the literature across PubMed, Cochrane Library, and EMBASE to identify randomized controlled trials (RCTs) evaluating the effect of thermal-softened ETTs on postoperative sore throat in patients undergoing elective surgeries under GA. The primary outcome was postoperative sore throat incidence, while secondary outcomes included hoarseness, vocal cord lesions, and time to intubation. Data were extracted independently by two authors, and the risk of bias was assessed using the Revised Cochrane risk of bias tool (version 2.0). A meta-analysis was then performed using the random-effects model, with the results expressed as risk ratios (RRs) and mean difference (MDs). **Results**: Eight studies, with a total of 970 participants, were included. Thermal-softened ETTs significantly reduced postoperative sore throat incidence (RR: 0.60, 95% CI: 0.44 to 0.82, *p* = 0.001). Subgroup analysis showed no difference for single-lumen tubes (RR: 0.76, 95% CI: 0.45 to 1.26, *p* = 0.28), but remained significant for double-lumen tubes (RR: 0.5, 95% CI: 0.39 to 0.65, *p* < 0.00001). No significant difference was found in hoarseness (RR: 0.86, 95% CI: 0.64 to 1.17, *p* = 0.34), but a lower incidence of vocal cord lesions (RR: 0.52, 95% CI: 0.40 to 0.68, *p* < 0.00001) was observed. No difference was found in the time to intubation (MD: −6.51, 95% CI: −20.04 to 7.02, *p* = 0.35). **Conclusions**: Thermal-softened ETTs may reduce the incidence of postoperative sore throat and vocal cord lesions but have no significant effect on hoarseness or intubation time.

## 1. Introduction

Postoperative sore throat is a common complication following general anesthesia and endotracheal intubation. The reported incidence of postoperative sore throat can be up to 62% [1], causing a reduction in patient satisfaction and a delay in postoperative recovery [2]. The etiology of postoperative sore throat is reported to be associated with the mechanical trauma caused by the insertion of the endotracheal tube (ETT), which can lead to irritation, inflammation, or injury of the airway mucosa [3]. In contrast, postoperative hoarseness and vocal cord lesions, although also common, are objective in nature and more challenging to assess, often relying on clinical tests for diagnosis. The reported incidence of postoperative hoarseness can range from 19.7% to 55% [3,4,5], while vocal cord lesions occur in about 27% of cases [6]. Since the use of ETTs is essential for airway management during surgery, efforts to minimize the occurrence and severity of postoperative sore throat, hoarseness, and vocal cord injuries have led to various modifications in pharmacologic [7,8] and non-pharmacologic interventions [9,10]. Among the studies on non-pharmacological strategies to reduce the incidence and severity of postoperative sore throat, approaches have included preintubation gargling with sodium azulene sulfonate or licorice, inflating the ETT cuff with normal saline, post-extubation cold vapor therapy, and gargling with honey lemon water or green tea. One such modification involves the use of thermal-softened ETTs, which are prewarmed before insertion to reduce the mechanical and thermal irritation caused by the cold, rigid tubes traditionally used in clinical practice. The rationale behind this intervention is based on the idea that heating the ETT may make it more flexible, thereby decreasing the force exerted on the airway during intubation and minimizing the risk of trauma to the mucosal surfaces of the trachea and larynx.

Despite the theoretical benefits, the clinical evidence regarding the effectiveness of thermal-softened ETTs in reducing postoperative sore throat and its related complications remains inconclusive. Some studies suggest a positive impact on reducing these complications, while others report no significant benefit. This systematic review and meta-analysis aims to primarily evaluate the effect of thermal-softened ETTs on the incidence of postoperative sore throat, and the secondary outcomes include the incidence of hoarseness, incidence of vocal cord lesions, and time to intubation. By critically assessing the data from randomized controlled trials (RCTs), this study seeks to provide a clearer picture of whether this modification in ETT preparation is a beneficial intervention for patients undergoing elective surgeries under general anesthesia.

## 2. Materials and Methods

This study was registered with the International Prospective Register of Systematic Reviews (PROSPERO, CRD42024597508). We reported the findings of the study following the Preferred Reporting Items for Systematic Reviews and Meta-analysis (PRISMA) guidelines [11]. Ethical approval was not required for this study.

### 2.1. Eligibility Criteria

#### 2.1.1. Participants

This study included randomized control trials (RCTs) evaluating the effect of thermal-softened ETT on postoperative sore throat in patients undergoing elective surgeries under general anesthesia (GA). The participants of the included trials were adults (≥18 years old) without a prior history of throat discomfort nor lesions of the vocal cords. Single-lumen (SLT) and double-lumen endotracheal tubes (DLT) were included. We excluded studies using supraglottic airways, case reports, animal experiments, systematic reviews, observational studies, and those where the full text was unobtainable.

#### 2.1.2. Interventions

The interventions included in this study were endotracheal tubes immersed in warmed saline before intubation. For the DLTs, the immersed portion consisted of the bronchial tip to the proximal margin of the tracheal cuff; for the SLTs, the distal portion was immersed.

#### 2.1.3. Comparators

The comparators were ETTs prepared at room temperature.

#### 2.1.4. Outcomes

The primary outcome of our study was the incidence of postoperative sore throat. The secondary outcomes were the incidence of postoperative hoarseness, incidence of vocal cord lesions, and intubation time.

### 2.2. Search Strategy and Study Selection

A search of PubMed, Cochrane, and EMBASE for relevant publications until 31 January 2025 was conducted by two authors (Hui-Zen Hee and Chen-Hsi Chiu), with the search limited to studies published in English. We used MeSH terminology and free-text keywords related to ‘thermal softening’, ‘endotracheal tubes’, ‘sore throat’ and ‘randomized controlled trial (RCT)’. The full search strategies for all databases are presented in the Supplementary Material Table S1. We used the “related articles” option in PubMed to extend the search scope and reviewed all retrieved abstracts, studies, and citations. We also reviewed the references of the relevant papers to retrieve related trials. To identify ongoing or unpublished studies, we also searched ClinicalTrials.gov and WHO ICTRP databases. PROSPERO, an online international prospective register of systematic reviews curated by the National Health Service, United Kingdom, has accepted our review protocol (file number: CRD42024597508).

### 2.3. Data Extraction and Methodological Quality Appraisal

The details of study designs, sample characteristics, inclusion and exclusion criteria, and outcome data of the included trials were extracted by two reviewers (Hui-Zen Hee and Chen-Hsi Chiu). Using a standardized form, the reviewers independently extracted relevant information from all eligible studies. The data items to be extracted included the following: (i) study characteristics (author names, year of publication, country where participants were enrolled), (ii) patient characteristics (age, gender, smokers, pre-existing throat condition), (iii) details of surgical procedure (type of surgery, type of intubating tubes, method of intubation), (iv) details of intervention and comparisons (temperature of saline used for tube immersion, duration of immersion), and (v) information on outcomes of interest. The data extracted were tabulated in Excel files and later analyzed. After that, the two reviewers evaluated the methodological quality of each randomized control trial according to the revised Cochrane risk of bias tool for randomized trials (version 2.0, released on 22 August 2019). Any disagreements were discussed and consulted with the senior reviewer (Cheng-Wei Lu) and resolved afterwards.

### 2.4. Statistical Analysis

Data were analyzed with RevMan Web. The effect size of dichotomous outcomes was analyzed using the risk ratio (RR) and the continuous outcomes using mean difference (MD). The precision of the effect size was reported as a 95% confidence interval.

The random-effects model was applied for all analysis, given the anticipated variability between studies. This approach was selected to account for potential differences in study designs, participant characteristics, and interventions across the included trials. Statistical heterogeneity across studies was assessed using the I^2^ statistics, with values greater than 50% suggesting substantial heterogeneity. Subgroups were explored with a consideration of the effect of thermal softening on SLTs versus DLTs. The test for subgroup differences was performed using the Chi^2^ test to evaluate the significance of the differences between subgroups.

### 2.5. Subgroup and Sensitivity Analysis

Sensitivity analysis was conducted to evaluate the robustness of the findings. We repeated the meta-analysis after sequentially excluding individual studies, particularly those with a disproportionate influence on the overall effect size, or those contributing substantially to heterogeneity. The goal was to determine whether the direction and statistical significance of the pooled effect would remain consistent.

Subgroup analyses were performed to compare the effect of thermal-softened endotracheal tubes between SLTs and DLTs, using the Chi^2^ test for subgroup differences, as implemented in RevMan Web. Additional subgroup analysis comparing nasal versus oral intubation was also conducted.

### 2.6. Assessment of Publication Bias

Publication bias was assessed for the primary outcome, the incidence of postoperative sore throat. A funnel plot was visually inspected to evaluate potential asymmetry in the distribution of the study effect sizes relative to their standard errors. Additionally, Egger’s regression test was performed using the metabias( ) function from the R meta package to statistically assess funnel plot asymmetry. A *p*-value less than 0.05 was considered to suggest that smaller studies might show systematically different results from larger studies, which could indicate the presence of publication bias or other small-study effects. Secondary outcomes were not assessed for publication bias due to inconsistent reporting across the included studies.

Our search for ongoing or unpublished trials yielded 3 trials without available data. To assess the potential impact of these unpublished studies on publication bias, we conducted a sensitivity analysis by simulating the hypothetical data. Details of the analysis are attached in the Supplementary Material Document S1.

### 2.7. Certainty of Evidence (GRADE Assessment)

The certainty of the evidence for each outcome was assessed using the Grading of Recommendations Assessment, Development, and Evaluation (GRADE) [12] approach. Certainty was rate as high, moderate, low, or very low, based on the five domains: risk of bias, inconsistency, indirectness, imprecision, and publication bias. Two reviewers (Hui-Zen Hee and Chen-Hsi Chiu) independently rated the certainty of evidence for the primary and secondary outcomes, and disagreements were resolved by a senior reviewer (Cheng-Wei Lu).

## 3. Results

### 3.1. Study Selection

**Figure 1:**
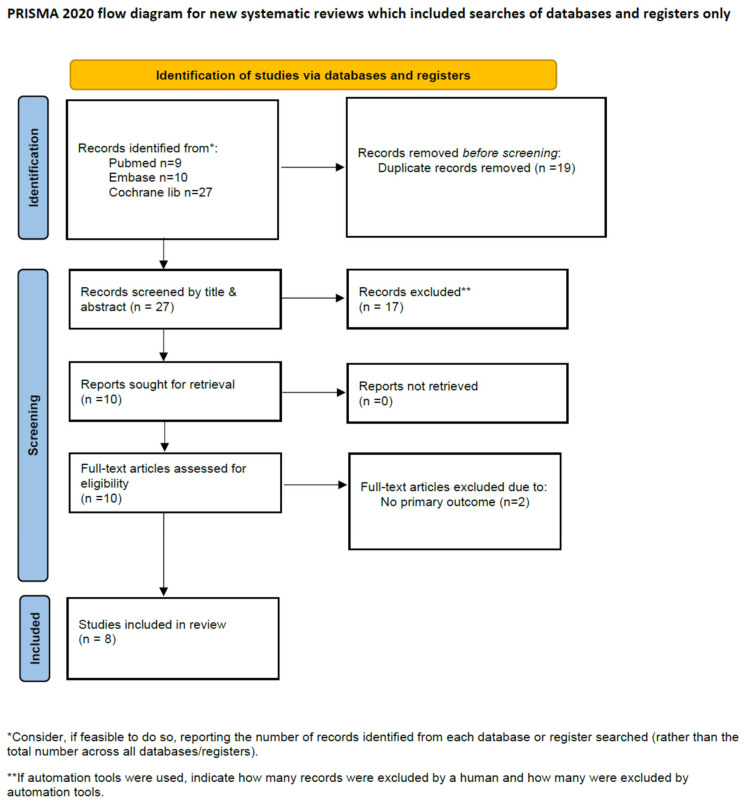
PRISMA flow diagram.

The search and selection processes are shown in Figure 1. The initial search of the literature yielded 46 articles, of which 19 were excluded after the initial review due to duplications and language. After scanning the titles and abstracts, 17 were further excluded, leaving 10 articles for full-text assessment. After full-text assessment, the studies by Yu [13] and Kim [14] were excluded due to having no primary outcome; eight studies met the inclusion criteria and were included in our final review.

### 3.2. Characteristics of the Included Studies

Table 1 summarizes the characteristics of the eight included trials. They were published between 2013 and 2023, with sample sizes ranging from 58 to 258 subjects.

#### 3.2.1. Intubation Methods

Among these trials, two recruited patients who underwent blind nasal intubation [15,16], three used single-lumen orotracheal tubes [17,18,19], and three employed double-lumen tubes [20,21,22]. The intubation methods by those who reported included direct laryngoscopy and video-laryngoscopy, and the study by Mohseni et al. [17], did not specify the tools used for intubation.

#### 3.2.2. Cuff Pressure Management

Reviewing previous studies, cuff pressure is one of the contributors to postoperative sore throat [23,24]. Hence, we reviewed the methods of cuff inflation for our eight included studies and found for the single-lumen tubes, the cuffs were inflated with pressures reported between 20–25 cmH_2_O or by auscultation until no leak was heard. For the DLTs, the tracheal cuff pressures were maintained below 25 cmH_2_O and the bronchial cuff pressures below 44 cmH_2_O. The two studies investigating blind nasal intubation did not mention the cuff pressures.

#### 3.2.3. Participant Demographics and Inclusion Criteria

The mean age of the participants ranged from 18 to 75 years. While most of the studies excluded smokers and patients with throat problems, one study specifically recruited patients with prior SARS-CoV infection [22], and another specifically recruited smokers [20]. Notably, Mohseni et al. [17] included patients with a stated criteria such as surgery on the head and chest, clear hoarseness, previous laryngeal surgery, abnormal position during the operation, sore throat for any reason, history of reflux or use of anti-reflux drugs, and smoking. However, in the table of their reported results categorized by surgery types, no surgeries involving the head and chest were reported. We hypothesize that these inclusion criteria were likely a typographical error and should have referred to exclusion criteria, as it would be inconsistent with the study design to include patients with baseline throat issues when examining the effect of thermal softening on postoperative sore throat. We reached out to the authors for clarifications, but regretfully we did not receive a reply.

#### 3.2.4. Use of Adjunctive Medications

As previous studies suggest that premedication with lidocaine [25] and dexamethasone [26,27] might contribute to reducing the incidence of postoperative sore throat, we reviewed the included studies and found that Mohseni et al. routinely used lidocaine [17], while Pasha et al. routinely used dexamethasone [16]. In the study by Yu et al., dexamethasone was used, but not routinely. However, the authors reported that the incidence of dexamethasone use did not differ between the thermal softening group and control groups [19].

#### 3.2.5. Thermal Softening Protocols

The thermal softening methods implemented in the trials included the immersion of the endotracheal or endobronchial tube in 40 or 50 degree Celsius warm saline for 5 to 10 min. Interestingly, the study by Song et al. [18] categorized the participants into four groups: control group, endotracheal tube lubricated with lidocaine group, thermal-softened endotracheal tube group, and thermal-softened endotracheal tubes lubricated with lidocaine group. For more consistent results and a clearer representation, we only extracted data from the control group and the thermal-softened endotracheal tube group.

**Table 1:** Characteristics of the included studies.

Study	Inclusion Criteria	No. of Patients (Male)	Age	Intubation Method	Types of Tube	Cuff Pressure	Premedication with Dexamethasone or Lidocaine	Intervention
Bi et al., 2022 [20]	Smokers for >5 years One-lung anesthesia surgery	C = 129 (100) TS = 129 (97)	>18	Direct laryngoscope	PVC DLT	Tracheal: <25 cmH_2_O Bronchial: <44 cmH_2_O	No	40 °C NS for 10 min
Hosseinzadeh et al., 2013 [15]	Elective maxillofacial surgery	C = 30 TS = 30	15–65	Blind nasal intubation	Nasal tube	n/a	No	50 °C for 5 min
Mohseni et al., 2022 [17]	Elective surgery except for head and chest surgery	C = 29 (12) TS = 29 (13)	19–69	N/A	SLT	Inflate until no leak	Lidocaine	40 °C for 7–8 min
Pasha et al., 2015 [16]	Elective maxillofacial surgery	C = 40 (25) TS = 40 (21)	18–65	Blind nasal intubation	Nasal tube	n/a	Dexamethasone	50 °C for 5 min
Seo et al., 2016 [21]	Elective thoracic surgery under one-lung anesthesia	C = 70 (37) TS = 70 (40)	20–75	Direct laryngoscope	PVC DLT	Tracheal: <25 cmH_2_O Bronchial: <44 cmH_2_O	No	40 °C for 10 min
Song et al., 2019 [18]	Elective laparoscopic cholecystectomy or ovarian cystectomy	C = 34 (12) L = 33 (11) TS = 35 (10) TS+L = 34 (8)	20–70	Direct laryngoscope	SLT	20–25 cm H_2_O	No	L: 2% lidocaine jelly TS: 40 °C for 5 min
Yan et al., 2023 [22]	Prior SARS CoV-2 3 months prior to surgery Thoracoscopic lung surgery	C = 60 (31) TS = 60 (33)	18–75	Video-laryngoscope	PVC DLT	Tracheal: <25 cmH_2_O Bronchial: <44 cmH_2_O	No	50 °C saline for 10 min
Yu et al., 2021 [19]	Elective nose surgery	C = 91 (52) TS = 94 (70)	>18	Video-laryngoscope	Oral RAE	20–25 cmH_2_O	Dexamethasone	40 °C saline for 10 min

C: control group, endotracheal tube prepared at room temperature; TS: thermal softening group, endotracheal tube immersed in warm saline before intervention; L: endotracheal tube lubricated with lidocaine; SLT: single-lumen tube; DLT: double-lumen tube; RAE: preformed Ring–Adair–Elwyn tube; NS: normal saline.

### 3.3. Risk of Bias Assessment

**Figure 2:**
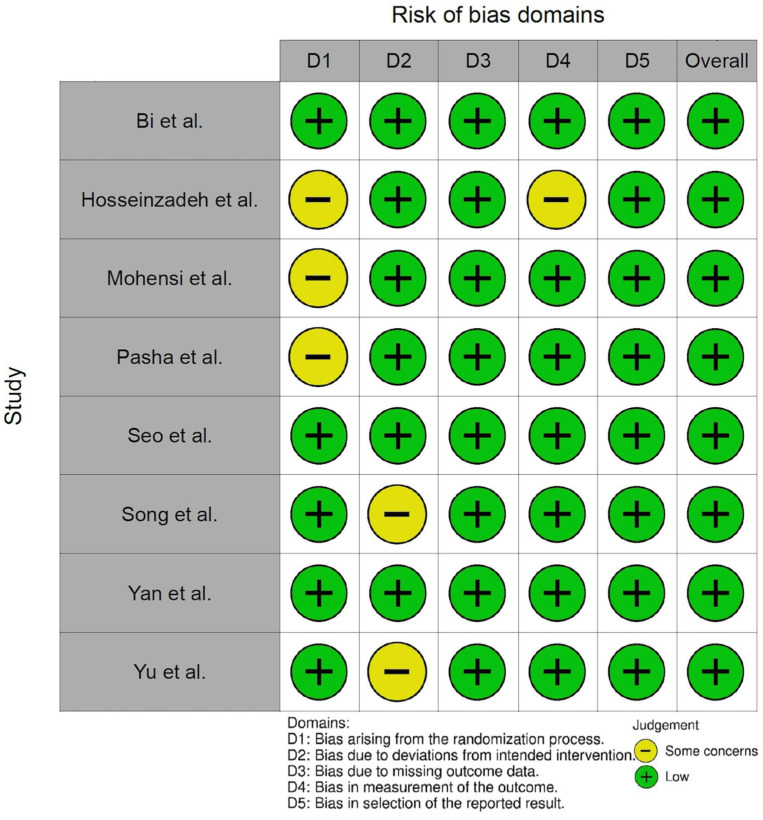
Risk of bias plot [15,16,17,18,19,20,21,22].

Two reviewers, Hui-Zen Hee and Chen-Hsi Chiu, independently assessed the risk of bias for each outcome of interest in each trial using the Cochrane risk of bias tool (RoB 2.0) [28]. Five domains were assessed, as follows: (1) bias from the randomization process, (2) bias due to deviations from the intended interventions, (3) bias due to missing outcome data, (4) bias in the measurement of the outcome, and (5) bias in the selection of the reported results. We used the robvis tool [29] to create the risk of bias plots. The risk of bias plot in Figure 2 summarizes the quality assessment of the included trials. Any disagreements were resolved by senior reviewer, Cheng-Wei Lu, afterwards.

### 3.4. Primary Outcome: Postoperative Sore Throat

#### 3.4.1. Pooled Analysis

In the included studies, sore throat occurrences were documented at various postoperative time intervals. For the purposes of this analysis, data for postoperative sore throat were extracted at postoperative day 1, or 24 h postoperatively. In the studies conducted by Hosseinzadeh et al. [15] and Pasha et al. [16], the specific timing of postoperative sore throat assessment and methods used to measure this outcome were not clearly stated; therefore, we assumed a 24 h postoperative period for consistency. In the remaining studies, postoperative sore throat was assessed through verbal questioning by investigators who were blinded to group allocation. These assessors inquired not only about the presence of sore throat but also about the severity of discomfort. To ensure consistency in data extraction, we only included the reported incidence of postoperative sore throat as a binary outcome (presence or absence).

Our pooled analysis demonstrated that the application of thermally softened ETTs reduced the incidence of postoperative sore throat when compared to room-temperature tubes (RR: 0.60, 95% CI: 0.44 to 0.82, *p* = 0.001). Heterogeneity across studies was moderate (I^2^: 45%, Chi^2^: 12.64, df:7, *p* = 0.008).

#### 3.4.2. Subgroup Analyses

However, when the data were further stratified to SLTs and DLTs, the effect of thermal softening varied between the two groups. For SLTs, thermal softening did not show a significant effect on reducing postoperative sore throat (RR:0.76, 95% CI: 0.45 to 1.26, *p* = 0.28). In this subgroup, the I^2^ statistic was 34%, indicating moderate heterogeneity among the studies. However, the corresponding *p*-value for heterogeneity was 0.2 (Chi^2^ = 6.02, df = 4), suggesting that the observed variability in effect sizes was not statistically significant.

**Figure 3:**
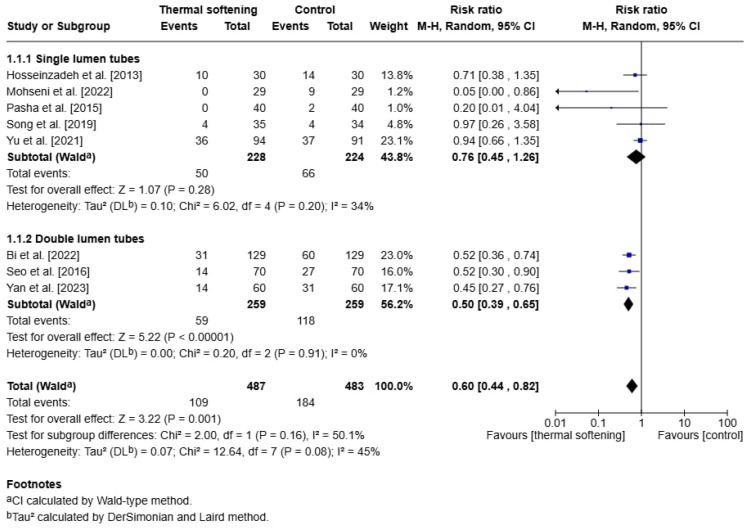
Forest plot of the incidence of postoperative sore throat on postoperative day 1 or 24 h postoperatively [15,16,17,18,19,20,21,22].

In contrast, for DLTs, thermal softening significantly reduced the incidence of postoperative sore throat (RR:0.5, 95% CI: 0.39 to 0.65, *p* < 0.00001). There was no heterogeneity in this subgroup (I^2^ = 0%, Chi^2^ = 0.2, df = 2, *p* = 0.91), indicating consistent findings across studies in this group. Tests for subgroup differences showed a non-significant difference (*p* = 0.16) (Figure 3).

Another supplementary subgroup analysis was conducted to explore the potential influence of intubation route (oral vs. nasal) among SLTs on the effectiveness of thermal softening in reducing postoperative sore throat. DLTs were excluded from this analysis, as they were structurally and functionally distinct and were inserted exclusively via the oral route. The comparison between oral (*n* = 3) and nasal (*n* = 2) SLT subgroups revealed a risk ratio of 0.67 (95% CI 0.22 to 2.01, *p* = 0.47) for oral intubation, and a risk ratio of 0.69 (95% CI 0.36 to 1.26, *p* = 0.22) for nasal intubation. There was no significant difference between subgroups (Chi^2^ = 0.00, *p* = 0.98, I^2^ = 0%) (Supplementary Material Figure S1).

#### 3.4.3. Sensitivity Analysis

Sensitivity analyses were performed by excluding key studies to assess the robustness of the primary outcome. After excluding Bi et al. [20] or Yu et al. [19], the largest studies in each subgroup, the pooled risk ratio remained statistically significant and continued to favor thermal-softened endotracheal tubes (RR: 0.62, 95%CI: 0.42 to 0.92 and RR: 0.53, 95% CI: 0.42 to 0.66, respectively). Additionally, the exclusion of Mohseni et al. [17]—a study with a large between-group event difference (0 vs. 9 events)—had a minimal impact on the overall result (RR: 0.62, 95%CI: 0.48 to 0.82). These findings suggest that the primary outcome was robust and not unduly influenced by any single study (Supplementary Material Figure S2).

#### 3.4.4. Publication Bias

**Figure 4:**
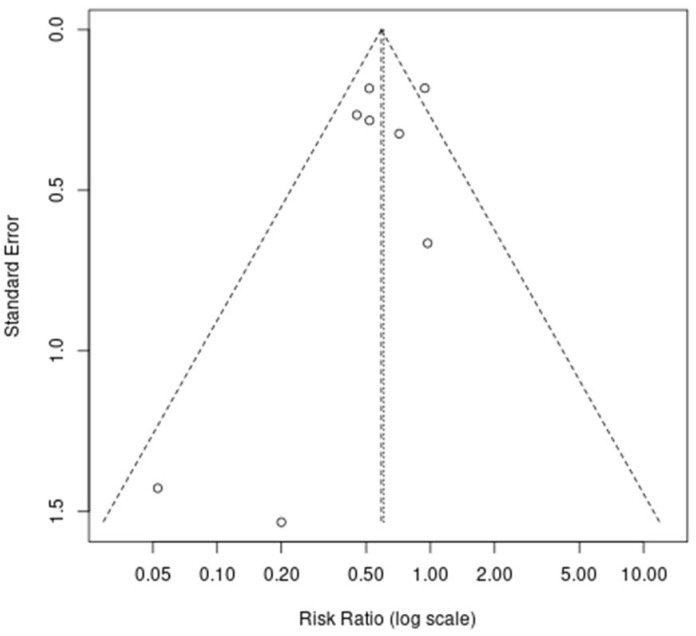
Funnel plot assessing publication bias for the incidence of postoperative sore throat. Studies are plotted by log risk ratio versus standard error. The vertical dotted line indicates the pooled effect estimate.

Publication bias was assessed using a visual inspection of a funnel plot and Egger’s regression test. As shown in Figure 4, the funnel plot appeared reasonably symmetrical, without clear evidence of asymmetry. Egger’s test yielded a bias coefficient of −1.02 (standard error = 0.89) with a *p*-value of 0.30. This result indicated no statistically significant small-study effects. However, as the total number of included studies *(n* = 8) was below the recommended minimum *(n* ≥ 10) for reliable asymmetry detection, these findings should be interpreted with caution.

An additional analysis for publication bias after including unpublished trials was performed. The results remained consistent across all tested scenarios, suggesting that the original finding of no significant publication bias was robust. Supplementary Material Document S1 presents the details of the analysis.

### 3.5. Secondary Outcome: Postoperative Hoarseness

#### 3.5.1. Pooled Analysis

Similarly, we extracted data for the incidence of postoperative hoarseness documented at postoperative day 1 or 24 h postoperatively. The studies by Bi et al. [20] and Pasha et al. [16] did not report this outcome and were therefore excluded from this analysis. In the overall analysis, the incidence of postoperative hoarseness did not differ between patients using thermal-softened ETTs and those who received room-temperature tubes (RR:0.86, 95% CI: 0.64 to 1.17, *p* = 0.34), with low heterogeneity across the studies (I^2^ = 11%).

#### 3.5.2. Subgroup Analysis

When stratified by tube type, the effect remained insignificant in both groups. For SLTs, the pooled RR was 0.50 (95% CI: 0.16 to 1.53, *p* = 0.22), with moderate heterogeneity (I^2^ = 48%). This variability may have reflected differences in sample sizes, intubation methods, or types of tubes (e.g., oral vs. nasal tubes). For DLTs, the pooled RR was 0.90 (95% CI: 0.62 to 1.31, *p* = 0.58, I^2^ = 0%).

**Figure 5:**
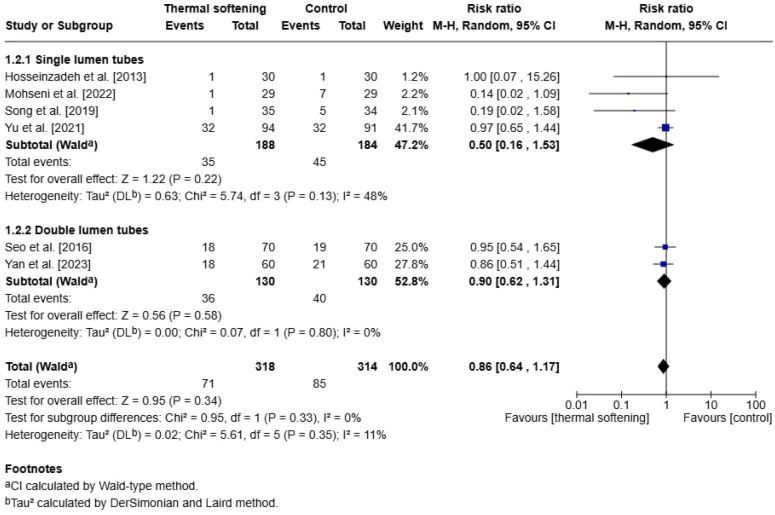
Forest plot of the incidence of postoperative hoarseness on postoperative day 1 or 24 h before sensitivity analysis [15,17,18,19,21,22].

Additionally, the test for subgroup differences did not reveal a significant effect (Chi^2^ = 0.95, df = 1, *p* = 0.33), and no significant heterogeneity was detected across the studies (I^2^ = 0%) (Figure 5).

#### 3.5.3. Sensitivity Analysis

**Figure 6:**
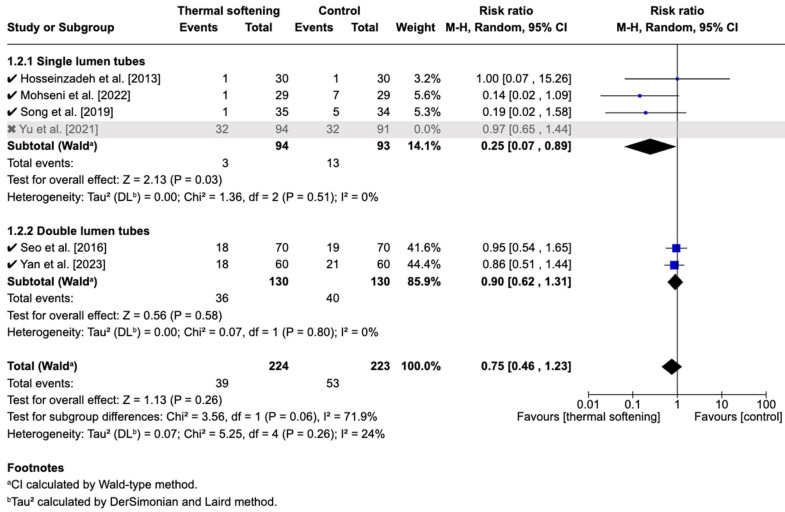
Forest plot of the incidence of postoperative hoarseness on postoperative day 1 or 24 h after sensitivity analysis [15,17,18,19,21,22]. ✓ indicates studies included in the analysis, x indicates study excluded due to large weight.

To assess the robustness of the findings, a sensitivity analysis was conducted by excluding the study by Yu et al. [19], which contributed the largest weight (41.7%) to the pooled effect estimate. After exclusion, the pooled risk ratio for SLTs became 0.25 (95% CI: 0.07 to 0.89, *p* = 0.03), suggesting a statistically borderline-significant reduction in postoperative hoarseness (Figure 6). This contrasted with the original non-significant finding, suggesting that the inclusion of Yu et al. may have attenuated the observed benefit of thermal-softened SLTs.

Furthermore, the test for subgroup differences following the exclusion of Yu et al. yielded an I^2^ of 71.9%, indicating substantial heterogeneity between the SLT and DLT subgroups. This suggests that thermal softening may have a stronger effect in SLTs than in DLTs in terms of reducing hoarseness. However, since this finding emerged from post hoc analysis with fewer studies, it should be interpreted with caution.

An additional analysis was conducted to evaluate the influence of the intubation route on the incidence of postoperative hoarseness, excluding studies that used DLTs, as these differed substantially in design and insertion method. Among the remaining four studies using SLTs, only Hosseinzadeh et al. [15] employed the nasal intubation route. A formal subgroup analysis was not feasible due to the limited number of nasal intubation studies. Instead, we conducted a sensitivity analysis by excluding the Hosseinzadeh study. The results remained consistent (RR: 0.40, 95% CI: 0.09 to 1.71, *p* = 0.21), suggesting that nasal intubation had a limited influence on the overall findings (Supplementary Material Figure S3).

### 3.6. Secondary Outcome: Vocal Cord Lesions

**Figure 7:**
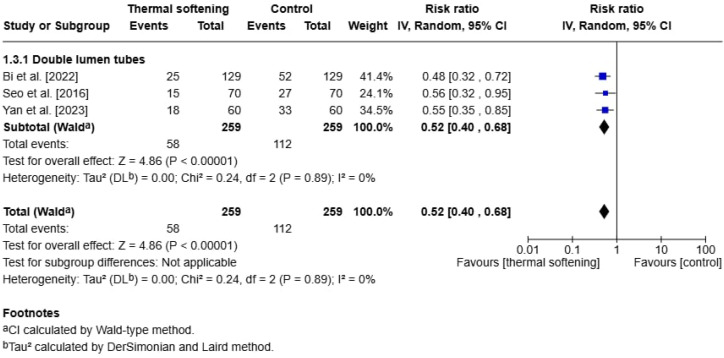
Forest plot of the incidence of vocal cord lesions on postoperative day 1 or 24 h postoperatively [20,21,22].

For another outcome of interest, vocal cord lesions after extubation, only three studies reported results [20,21,22]. All of these studies used DLTs. Post-extubation vocal cord lesions were assessed by blinded otorhinolaryngologists via fiberoptic bronchoscopes in each study. Additionally, preintubation examinations of vocal cords were also conducted in all three studies for comparison purposes. In the studies by Bi et al. [20] and Seo et al. [21], recorded vocal cord lesions consisted of petechia, edema, and hematoma; whereas Yan et al. [22] reported edema, erythema, and hematoma. In the overall analysis, the incidence of vocal cord lesions in patients using thermal-softened ETTs were significantly lower than those who received room-temperature tubes (RR:0.52, 95% CI: 0.40 to 0.68, *p* < 0.00001). The analysis showed no evidence of heterogeneity, with I^2^ = 0% and a Chi^2^ statistic of 0.24 (*p* = 0.89), suggesting that the included studies were highly consistent (Figure 7). Sensitivity analysis was not performed for this outcome due to the limited number of studies (*n* = 3) and the absence of heterogeneity.

### 3.7. Secondary Outcome: Time to Intubation

#### 3.7.1. Pooled Analysis

We also analyzed the effect of the thermal softening of ETTs on the time to intubation. The studies by Mohseni et al. [17] and Song et al. [18] did not report this outcome. In the remaining studies, the unit of measurement for time was uniformly seconds, hence mean difference (MD) was used for analysis. The overall analysis showed that there were no significant effects of the thermal softening of ETTs on the time to intubation (MD: −6.51, 95% CI: −20.04 to 7.02, *p* = 0.35).

#### 3.7.2. Subgroup Analysis

**Figure 8:**
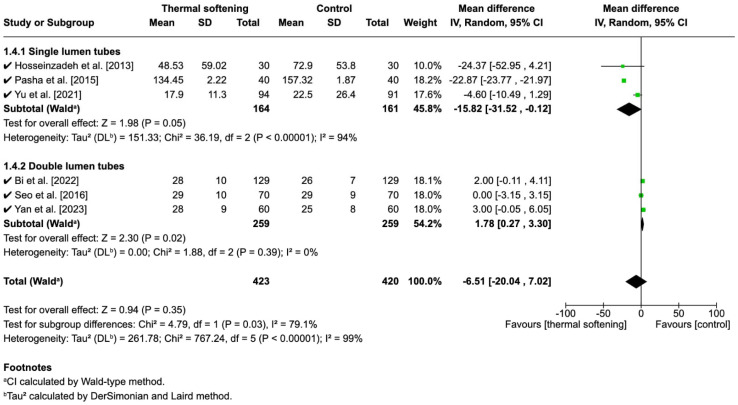
Forest plot of the time to intubation before sensitivity analysis [15,16,19,20,21,22]. ✓ indicates studies included in the analysis.

When stratified into subgroups, time to intubation was shorter for thermal-softened ETTs than for room-temperature tubes in the SLTs with borderline significance (MD: −15.82, 95% CI −31.52 to −0.12, *p* = 0.05), and were significantly longer in DLTs (MD:1.78, 95% CI: 0.27 to 3.30, *p* = 0.02) (Figure 8). However, the overall heterogeneity was high (I^2^ = 99%), suggesting significant variability across studies, which was mainly due to heterogeneity in the SLT group (I^2^ = 94%).

#### 3.7.3. Sensitivity Analysis

To address this, a sensitivity analysis was performed by excluding the study by Yu et al. [19], which contributed to the observed heterogeneity. This decision was based on differences in the intubation techniques: the method of intubation by Yu et al. was via video-laryngoscopy, while Hosseinzadeh et al. [15] and Pasha et al. [16] used blind nasal intubation. After excluding this study, the results for the SLT subgroup became more consistent, with a statistically significant reduction in time to intubation (MD: −22.87, 95% CI: −23.77 to −21.97, *p* < 0.00001). Importantly, the heterogeneity in this subgroup was resolved, with I^2^ reduced to 0%.

**Figure 9:**
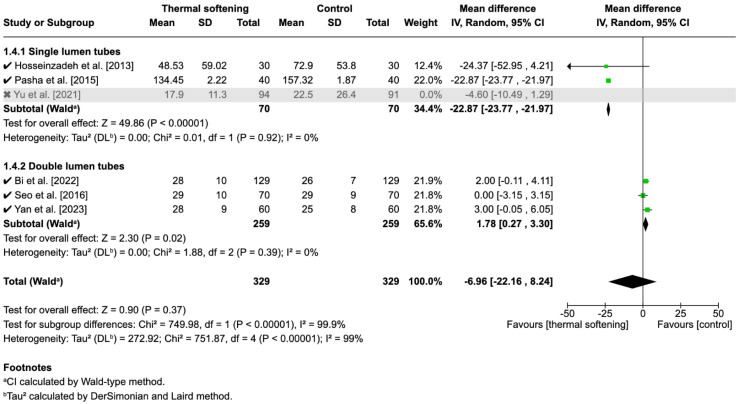
Forest plot of the time to intubation after sensitivity analysis [15,16,17,20,21,22]. ✓ indicates studies included in the analysis, x indicates study excluded due to different intubation technique.

Despite the significant findings in the SLT subgroup post-sensitivity analysis, the overall meta-analysis still showed no significant effect when combining both subgroups (MD: 6.96, 95% CI: −22.16 to 8.24, *p* = 0.37), with high heterogeneity across studies (I^2^ = 99%). A test for subgroup differences revealed a highly significant difference between the SLT and DLT subgroups (Chi^2^ = 749.98, *p* < 0.00001), further supporting the idea that the effects of thermal softening may differ based on tube type. The heterogeneity within the overall analysis was largely driven by the differences between the subgroups (Figure 9).

### 3.8. Summary of Certainty of Evidence

Using the GRADE framework, the certainty of evidence varied across outcomes. The summary of the findings is shown in Table 2. Evidence for reducing postoperative sore throat was rated as high, with no downgrade despite there being moderate heterogeneity (I^2^ = 45%), as this was explained by subgroup differences. Evidence for hoarseness was rated moderate, and downgraded for imprecision as the confidence interval crossed the line of no effect. Vocal cord lesion outcomes showed a consistent and precise benefit, rated as high certainty. For time to intubation, evidence was rated low due to imprecision and inconsistency.

## 4. Discussion

In this study, we evaluated whether thermal softening of endotracheal tubes could reduce laryngeal complications, including postoperative sore throat, hoarseness, and vocal cord lesions following general anesthesia with endotracheal intubation. While we recognize that several systematic reviews and meta-analyses have already investigated methods to prevent postoperative sore throat, our study contributes additional value by specifically focusing on the impact of the ETT temperature modification—an area that remains underrepresented in the existing literature.

### 4.1. Postoperative Sore Throat

The results of our analysis suggest that the use of thermally softened ETTs significantly reduced the incidence of postoperative sore throat compared to room-temperature ETTs. Specifically, thermal softening showed a marked benefit in patients with double-lumen tubes (RR = 0.50, *p* < 0.00001), but did not significantly reduce postoperative sore throat in the SLT subgroup (RR = 0.76, *p* = 0.28). Interestingly, the test for subgroup differences between these two groups did not show a statistically significant difference (*p* = 0.16), suggesting that the effect of thermal softening may not be substantially different between the two tube types. One possible explanation for the lack of significant effect in the SLT subgroup could be the moderate heterogeneity within this subgroup (I^2^ = 34%). This suggests that variability in the studies included in this subgroup might have contributed to this lack of statistical significance, rather than indicating that thermal softening had no effect on postoperative sore throat for SLTs. The heterogeneity in this group might have been due to the different methods of intubation, types of surgery, premedications (Table 1), as well as other factors that were not reported in those studies.

Postoperative sore throat is influenced by multiple factors, with predictors such as female sex, younger age, higher ASA class, pre-existing lung disease, prolonged anesthesia duration, duration of postoperative stay, intubation without neuromuscular blockade, the use of succinylcholine, the use of double-lumen tubes, increased tracheal tube cuff pressures, and types of procedures [1,30,31]. In contrast, the pronounced effect seen in DLTs could be attributed to the inherent characteristics of the DLT, which is typically larger in diameter and may exert more mechanical pressure on the airway, increasing the likelihood of mucosa trauma and, consequently a higher incidence of postoperative sore throat [32]. This finding may have clinical implications. When treating patients with the aforementioned risk factors who require one-lung ventilation, the use of SLT with a bronchial blocker could be a better choice for minimizing postoperative sore throat. However, the effect of a bronchial blocker on postoperative sore throat is a separate issue that warrants further investigation and discussion.

### 4.2. Hoarseness

In contrast to sore throat, the incidence of postoperative hoarseness initially showed no significant difference between patients using thermal-softened and room-temperature ETTs (RR: 0.86, 95% CI 0.64 to 1.17, *p* = 0.34). This result remained consistent across both SLT and DLT subgroups, suggesting that thermal softening may not provide a significant benefit in reducing hoarseness. While postoperative sore throat is mainly caused by inflammation of the airway tissues, hoarseness, on the other hand, can be associated with various mechanisms other than direct trauma to the vocal cords, such as recurrent laryngeal nerve palsy [33], arytenoid cartilage dislocation [34], or, in even rarer cases, Tapia’s syndrome [35]. This could partly explain how the benefit of thermally softened ETTs in reducing postoperative sore throat and vocal cord lesions may be more pronounced than in reducing hoarseness.

A sensitivity analysis excluding Yu et al. [19]—a study with a high weight and differing intubation method (video-laryngoscope)—revealed a statistically significant reduction in hoarseness within the SLT subgroup (RR: 0.25, 95% CI: 0.07 to 0.89, *p* = 0.03), suggesting a possible benefit that may have been obscured by heterogeneity in technique. This is consistent with the result of an analysis which revealed fewer incidences of hoarseness associated with video-laryngoscope intubation as compared to the traditional Macintosh blade [36]. From a clinical standpoint, this supports a more nuanced application of thermal softening: when video-guided laryngoscopy is not performed, softening the tubes may help minimize laryngeal irritation and hoarseness.

However, preventing hoarseness likely requires multifactorial strategies. Interventions beyond minimizing the mechanical trauma caused by intubation devices should be considered, such as locating the cuff more than 15 mm below the vocal cords to avoid the compression of the anterior branch of the recurrent laryngeal nerve [37], utilizing stylets for intubation [38], and meticulously positioning the head and neck to avoid excessive nerve stretching [39].

### 4.3. Vocal Cord Lesions

Another finding of our analysis is the reduction in vocal cord lesions, such as petechiae or edema and hematoma, when using thermal-softened DLTs (RR:0.52, 95% CI: 0.40 to 0.68, *p* < 0.00001). In our analysis, only those studies that used DLTs reported this outcome, and within this DLT group, there was no heterogeneity among studies, suggesting the consistent beneficial effect of thermal-softened DLTs in this aspect. These results offer compelling evidence for the adoption of this intervention in clinical practice, indicating that this approach may be widely effective in reducing the incidence of vocal cord lesions. Besides the thermal softening of the DLTs, published data by Seo et al. [40] suggest that a 180° rotation during DLT advancement through the glottis can also help reduce the risk of vocal cord injury. Meanwhile, the studies included in our analysis established an initial 90° rotation after endotracheal tube advancement through the glottis. Another factor that might contribute to vocal cord lesions during intubation is the intubation method. In a randomized control trial comparing video-laryngoscopy and direct laryngoscopy for double-lumen endotracheal tube intubation in thoracic surgery, the authors concluded that video-laryngoscopy reduced the incidence of hematoma, hemorrhage, and blood-tinged vocal cords [41]. Reviewing the studies in our analysis, two studies [20,21] used direct laryngoscopy and one [22] used video-laryngoscopy, but the differences in intubation method did not introduce significant heterogeneity across the studies. However, in our analysis, the studies that reported this outcome were only performed with DLTs, and further studies are needed to determine whether the same findings can be replicated with SLTs, potentially broadening the scope of clinical applications.

### 4.4. Time to Intubation

The overall analysis of time to intubation did not reveal a significant difference. However, the heterogeneity between SLTs and DLTs is high. Subgroup analysis indicated a borderline significant reduction in intubation time in the SLT group, albeit with high heterogeneity within this subgroup.

The SLT subgroup included three studies—two using blind nasal intubation [15,16] and one using a video-laryngoscope [19]. After excluding the study by Yu et al. [19] for sensitivity analysis, the SLT group showed a significant difference in terms of reduction in intubation time, with reduced heterogeneity. This should be interpreted with caution. Clinically, it suggests that routine thermal softening of endotracheal tubes may offer limited benefit in general practice. However, in cases involving blind nasal intubation, the observed absolute time difference up to 20 s, while seemingly modest, could be clinically meaningful. In situations where patients have limited oxygen reserves, prolonged intubation attempts, particularly with blind techniques, can substantially increase the risk of desaturation.

The previous literature suggested that the increased flexibility of the thermal-softened nasotracheal tubes may make them less navigable and therefore less suitable for blinded intubation [42]. Interestingly, our results showed that thermal-softened SLTs might be beneficial in shortening the intubation time. Additionally, thermal-softened nasotracheal tubes poses other advantages, such as reducing the incidence and severity of epistaxis [14]. Although the widespread use of fiberoptic scopes has led to a decline in blind nasal intubation as a commonly practiced technique, we believe that mastering this technique, along with its relevant modifications to minimize associated complications, still holds clinical value [43].

Alternatively, in the DLT subgroup, although individual studies did not show statistically significant differences in intubation time between thermal-softened and room-temperature tubes, the pooled analysis revealed a statistically significant effect. This can be explained by the nature of the meta-analysis itself, which increased its statistical power by pooling data from multiple studies. While each individual study may have limited power due to a smaller sample size or varying study design, the combined sample size from all three studies allowed for a more precise estimate of the overall effect [44]. Even small differences observed in larger studies, such as in the study by Bi et al. [20], can have a substantial impact on the pooled result, leading to statistical significance despite the absence of significant findings in the individual studies. Although the pooled analysis showed a statistically significant difference, this effect may not be clinically relevant in most cases, as the actual time difference was minimal (typically less than 3 s). Specifically, the mean time difference was only 1.78 s. It is important to avoid overinterpreting this result. One should not conclude that the thermal softening of DLTs always lead to prolonged intubation time, nor that the modification is without value.

### 4.5. Route of Endotracheal Intubation

Another potential confounder in our analysis was the route of endotracheal intubation (oral vs. nasal). A systematic review by El-Boghdadly et al. found that nasotracheal intubation might be associated with a higher incidence of postoperative sore throat compared to orotracheal intubation [1]. Furthermore, Tsukamoto et al. reported a notably high incidence of postoperative sore throat (74.6%) following nasotracheal intubation in oral and maxillofacial surgery [45]. To explore this factor, we conducted a subgroup analysis, only including studies that used SLTs, as DLTs were exclusively inserted via the oral route. Our results showed no significant effect of thermal softening in postoperative sore throat reduction when subgrouped into oral and nasal intubation routes (Supplementary Material Figure S1). However, this analysis was limited by the small number of studies that clearly employed nasal intubation (*n* = 2), and the findings should be interpreted with caution. Nonetheless, given that nasal intubation may carry a different risk profile for airway trauma compared to oral intubation, further research is warranted to clarify this potential confounder.

For postoperative hoarseness, a sensitivity analysis was conducted, again excluding DLT studies. As stated in the previous Methods section, we conducted a sensitivity analysis instead of a subgroup analysis due to limited number of nasal intubation studies for this outcome (*n* = 1). The sensitivity analysis, excluding this study, yielded consistent findings, indicating that nasal intubation had a limited influence on the pooled effect of thermal softening on postoperative hoarseness (Supplementary Material Figure S3).

Regarding vocal cord lesions, all contributing studies involved DLTs and oral intubation. As such, no further analysis based on intubation route was necessary for this outcome.

### 4.6. Limitations

There are several limitations that should be acknowledged. First, heterogeneity among the included studies may have influenced our conclusions. Differences in patient populations, surgical procedures, tube types, and intubation methods contributed to variability in our analyses. For clarity, we have listed the possible confounding factors in Table 1 to help readers interpret the results. Second, the number of studies included was limited, particularly for vocal cord lesions, where all available data were derived from studies using DLTs. As a result, the effect of thermal softening on vocal cord lesions in SLTs remains unclear. Third, potential confounding factors were not consistently reported across studies. These include premedication use (e.g., lidocaine or dexamethasone, cuff pressure management, saline temperature and soaking time, and the experience level of clinicians). These variables may have influenced the incidence of postoperative sore throat and other outcomes. Additionally, the exclusion criteria varied, with one study including patients with prior SARS-CoV-2 infection [22] and another including smokers [20], which may have limited the generalizability of our findings. Therefore, the results should be interpreted with caution, and further studies are needed to address these limitations and provide more definitive conclusions.

## 5. Conclusions

Based on the results of our systematic review and meta-analysis, thermally softened ETTs reduce the incidence of postoperative sore throat and the occurrence of vocal cord lesions, particularly for DLT intubation. However, this method did not significantly affect the reduction in postoperative hoarseness in either SLT or DLT intubation. On the other hand, the impact on intubation time showed borderline significance for SLTs, likely due to the high heterogeneity across studies, while it increased slightly for DLTs, with minimal clinical relevance. In conclusion, while the findings offered valuable insights, limitations such as the small number of studies in some subgroups and the variability across studies highlighted the need for more rigorous and consistent research to refine our understanding of these effects in clinical practice.

## Figures and Tables

**Table 2:** Summary of findings on thermal softening of endotracheal tubes across outcomes.

Outcomes	Number of Participants (Studies)	Certainty of the Evidence (GRADE)	Relative Effect (95% CI)	Anticipated Absolute Effects	
				Risk with Room-Temperature ETT	Risk Difference with Thermal-Softened ETT
Postoperative sore throat	969 (5 RCTs)	⬤⬤⬤⬤ High	RR 0.60 (0.44–0.82)	381 per 1000	152 fewer per 1000 (27 to 84 fewer)
Postoperative hoarseness	632 (4 RCTs)	⬤⬤⬤◯ Moderate ^a^	RR 0.86 (0.64–1.17)	271 per 1000	38 fewer per 1000 (97 fewer to 46 more)
Vocal cord lesions	518 (3 RCTs)	⬤⬤⬤⬤ High	RR 0.52 (0.40–0.68)	432 per 1000	208 fewer per 1000 (138 to 259 fewer)
Time to intubation	843 (4 RCTs)	⬤⬤◯◯ Low ^b^	MD −6.51 s (−20.04 to 7.02)	—	MD 6.51 s shorter

CI: confidence interval; MD: mean difference; RR: risk ratio; GRADE: Working Group grades of evidence; **GRADE symbols**: ● = one level of certainty; ●●●● = High; ●●●○ = Moderate; ●●○○ = Low; ●○○○ = Very low; High certainty: we are very confident that the true effect lies close to that of the estimate of the effect; Moderate certainty: we are moderately confident in the effect estimate; the true effect is likely to be close to the estimate of the effect, but there is a possibility that it is substantially different; Low certainty: our confidence in the effect estimate is limited; the true effect is likely to be substantially different from the estimate of effect; ^a^ downgraded for imprecision; ^b^ downgraded for imprecision and inconsistency.

## Data Availability

Data are contained within the article or Supplementary Materials. The original contributions presented in this study are included in the article/Supplementary Materials. Further inquiries can be directed to the corresponding author(s).

## Supplementary Materials

The following supporting information can be downloaded at: https://www.mdpi.com/article/10.3390/jcm14113620/s1, Table S1: Search strategy for record extraction. Document S1: Publication bias after including unpublished trials. Figure S1: Forest plot of incidence of postoperative sore throat after subgroup analysis based on route of intubation (oral vs. nasal). Figure S2: Forest plot of incidence of postoperative sore throat after sensitivity analysis. Figure S3: Forest plot of incidence of postoperative hoarseness after sensitivity analysis.

## Abbreviations

The following abbreviations are used in this manuscript:

ETT	Endotracheal tube
GA	General anesthesia
RCTs	Randomized controlled trials
RRs	Risk ratios
MDs	Mean differences
PROSPERO	Prospective Register of Systematic Reviews
PRISMA	Preferred Reporting Items for Systematic Reviews and Meta-analysis
SLT	Single-lumen endotracheal tube
DLT	Double-lumen endotracheal tube
