# Supplementary material for: The Effect of Thermal-Softened Endotracheal Tubes on Postoperative Sore Throat and Other Complications—A Systematic Review and Meta-Analysis
Source: J Clin Med. 2025 May 22;14(11):3620. doi: 10.3390/jcm14113620 (PMC12155181; doi:10.3390/jcm14113620)
Supplement: Supplementary file 1 [file jcm-14-03620-s001.zip › Supplementary Material Figure S2A-C .pdf]

Study or Subgroup	Thermal softening Events	Thermal softening Total	Control Events	Control Total	Weight	Risk ratio M-H, Random, 95% CI
**1.2.1 SLT**						
✔ Hosseinzadeh et al. [2013]	10	30	14	30	18.5%	0.71 [0.38 , 1.35]
✔ Mohseni et al. [2022]	0	29	9	29	1.8%	0.05 [0.00 , 0.86]
✔ Pasha et al [2015]	0	40	2	40	1.6%	0.20 [0.01 , 4.04]
✔ Song et al. [2019]	4	35	4	34	7.1%	0.97 [0.26 , 3.58]
✔ Yu et al. [2021]	36	94	37	91	28.1%	0.94 [0.66 , 1.35]
**Subtotal (Wald[a])**		**228**		**224**	**57.0%**	**0.76 [0.45 , 1.26]**
Total events:	50		66			
Test for overall effect: Z = 1.07 (P = 0.28)						
Heterogeneity: Tau^2 (DL[b], 95% CI) = 0.10 [0.00 , 11.84]; Chi^2 = 6.02, df = 4 (P = 0.20); I^2 = 34%						
**1.2.2 DLT**						
✖ Bi et al. [2022]	31	129	60	129	0.0%	0.52 [0.36 , 0.74]
✔ Seo et al. [2016]	14	70	27	70	20.9%	0.52 [0.30 , 0.90]
✔ Yan et al. [2023]	14	60	31	60	22.1%	0.45 [0.27 , 0.76]
**Subtotal (Wald[a])**		**130**		**130**	**43.0%**	**0.48 [0.33 , 0.70]**
Total events:	28		58			
Test for overall effect: Z = 3.78 (P = 0.0002)						
Heterogeneity: Tau^2 (DL[b], 95% CI) = 0.00 [0.00 , 9.64]; Chi^2 = 0.13, df = 1 (P = 0.72); I^2 = 0%						
**Total (Wald[a])**		**358**		**354**	**100.0%**	**0.62 [0.42 , 0.92]**
Total events:	78		124			
Test for overall effect: Z = 2.41 (P = 0.02)						
Test for subgroup differences: Chi^2 = 1.93, df = 1 (P = 0.16), I^2 = 48.2%						
Heterogeneity: Tau^2 (DL[b], 95% CI) = 0.10 [0.00 , 3.80]; Chi^2 = 11.20, df = 6 (P = 0.08); I^2 = 46%						

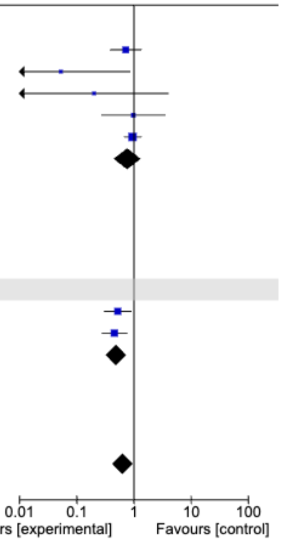

Supplementary Figure S2A: Forest plot of postoperative sore throat after sensitivity analysis (exclude Bi et al.)

Study or Subgroup	Thermal softening Events	Thermal softening Total	Control Events	Control Total	Weight	Risk ratio M-H, Random, 95% CI
**1.2.1 SLT**						
✔ Hosseinzadeh et al. [2013]	10	30	14	30	13.8%	0.71 [0.38 , 1.35]
✔ Mohseni et al. [2022]	0	29	9	29	0.7%	0.05 [0.00 , 0.86]
✔ Pasha et al [2015]	0	40	2	40	0.6%	0.20 [0.01 , 4.04]
✔ Song et al. [2019]	4	35	4	34	3.3%	0.97 [0.26 , 3.58]
✖ Yu et al. [2021]	36	94	37	91	0.0%	0.94 [0.66 , 1.35]
**Subtotal (Wald[a])**		**134**		**133**	**18.3%**	**0.53 [0.20 , 1.43]**
Total events:	14		29			
Test for overall effect: Z = 1.25 (P = 0.21)						
Heterogeneity: Tau^2 (DL[b], 95% CI) = 0.41 [0.00 , 23.35]; Chi^2 = 5.10, df = 3 (P = 0.16); I^2 = 41%						
**1.2.2 DLT**						
✔ Bi et al. [2022]	31	129	60	129	43.1%	0.52 [0.36 , 0.74]
✔ Seo et al. [2016]	14	70	27	70	18.0%	0.52 [0.30 , 0.90]
✔ Yan et al. [2023]	14	60	31	60	20.5%	0.45 [0.27 , 0.76]
**Subtotal (Wald[a])**		**259**		**259**	**81.7%**	**0.50 [0.39 , 0.65]**
Total events:	59		118			
Test for overall effect: Z = 5.22 (P < 0.00001)						
Heterogeneity: Tau^2 (DL[b], 95% CI) = 0.00 [0.00 , 0.18]; Chi^2 = 0.20, df = 2 (P = 0.91); I^2 = 0%						
**Total (Wald[a])**		**393**		**392**	**100.0%**	**0.53 [0.42 , 0.66]**
Total events:	73		147			
Test for overall effect: Z = 5.36 (P < 0.00001)						
Test for subgroup differences: Chi^2 = 0.01, df = 1 (P = 0.91), I^2 = 0%						
Heterogeneity: Tau^2 (DL[b], 95% CI) = 0.00 [0.00 , 3.17]; Chi^2 = 5.26, df = 6 (P = 0.51); I^2 = 0%						

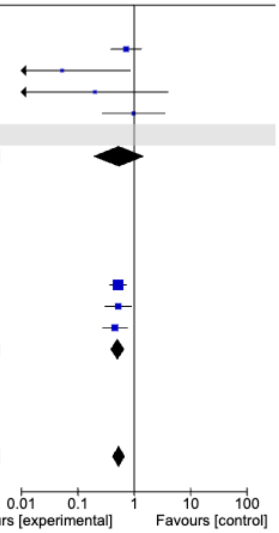

Supplementary Figure S2B: Forest plot of postoperative sore throat after sensitivity analysis (exclude Yu et al.)

Study or Subgroup	Thermal softening Events	Thermal softening Total	Control Events	Control Total	Weight	Risk ratio M-H, Random, 95% CI
**1.2.1 SLT**						
✔ Hosseinzadeh et al. [2013]	10	30	14	30	12.9%	0.71 [0.38 , 1.35]
✖ Mohseni et al. [2022]	0	29	9	29	0.0%	0.05 [0.00 , 0.86]
✔ Pasha et al [2015]	0	40	2	40	0.8%	0.20 [0.01 , 4.04]
✔ Song et al. [2019]	4	35	4	34	3.9%	0.97 [0.26 , 3.58]
✔ Yu et al. [2021]	36	94	37	91	25.0%	0.94 [0.66 , 1.35]
**Subtotal (Wald[a])**		**199**		**195**	**42.7%**	**0.87 [0.65 , 1.18]**
Total events:	50		57			
Test for overall effect: Z = 0.89 (P = 0.38)						
Heterogeneity: Tau^2 (DL[b], 95% CI) = 0.00 [0.00 , 6.00]; Chi^2 = 1.52, df = 3 (P = 0.68); I^2 = 0%						
**1.2.2 DLT**						
✔ Bi et al. [2022]	31	129	60	129	24.9%	0.52 [0.36 , 0.74]
✔ Seo et al. [2016]	14	70	27	70	15.5%	0.52 [0.30 , 0.90]
✔ Yan et al. [2023]	14	60	31	60	16.8%	0.45 [0.27 , 0.76]
**Subtotal (Wald[a])**		**259**		**259**	**57.3%**	**0.50 [0.39 , 0.65]**
Total events:	59		118			
Test for overall effect: Z = 5.22 (P < 0.00001)						
Heterogeneity: Tau^2 (DL[b], 95% CI) = 0.00 [0.00 , 0.18]; Chi^2 = 0.20, df = 2 (P = 0.91); I^2 = 0%						
**Total (Wald[a])**		**458**		**454**	**100.0%**	**0.62 [0.48 , 0.82]**
Total events:	109		175			
Test for overall effect: Z = 3.42 (P = 0.0006)						
Test for subgroup differences: Chi^2 = 7.53, df = 1 (P = 0.006), I^2 = 86.7%						
Heterogeneity: Tau^2 (DL[b], 95% CI) = 0.04 [0.00 , 0.55]; Chi^2 = 9.30, df = 6 (P = 0.16); I^2 = 35%						

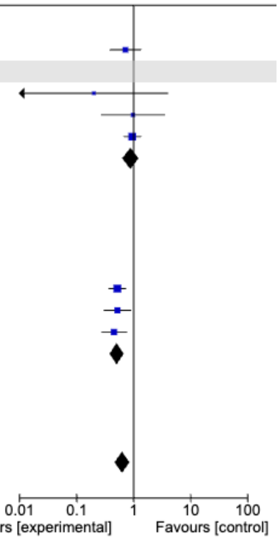

Supplementary Figure S2C: Forest plot of postoperative sore throat after sensitivity analysis (exclude Mohseni et al.)
