# Supplementary material for: The Effect of Thermal-Softened Endotracheal Tubes on Postoperative Sore Throat and Other Complications—A Systematic Review and Meta-Analysis
Source: J Clin Med. 2025 May 22;14(11):3620. doi: 10.3390/jcm14113620 (PMC12155181; doi:10.3390/jcm14113620)
Supplement: Supplementary file 1 [file jcm-14-03620-s001.zip › Supplementary Material Figure S3_ hoarseness sensitivity analysis.pdf]

Study or Subgroup	Thermal softening Events	Thermal softening Total	Control Events	Control Total	Weight	Risk ratio M-H, Random, 95% CI
**1.2.1 Single lumen tubes**						
✕ Hosseinzadeh et al. [2013]	1	30	1	30	0.0%	1.00 [0.07 , 15.26]
✓ Mohseni et al. [2022]	1	29	7	29	3.0%	0.14 [0.02 , 1.09]
✓ Song et al. [2019]	1	35	5	34	2.8%	0.19 [0.02 , 1.58]
✓ Yu et al. [2021]	32	94	32	91	38.6%	0.97 [0.65 , 1.44]
**Subtotal (Wald[a])**		**158**		**154**	**44.4%**	**0.40 [0.09 , 1.71]**
Total events:	34		44			

Test for overall effect: Z = 1.24 (P = 0.21)

Heterogeneity: Tau^2 (DL^b) = 1.09; Chi^2 = 5.75, df = 2 (P = 0.06); I^2 = 65%

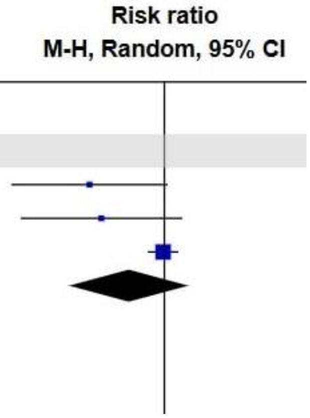

Supplementary Figure S3: Forest plot of postoperative hoarseness after sensitivity analysis (excluding Hosseinzadeh et al.)
