# Supplementary material for: The Effect of Thermal-Softened Endotracheal Tubes on Postoperative Sore Throat and Other Complications—A Systematic Review and Meta-Analysis
Source: J Clin Med. 2025 May 22;14(11):3620. doi: 10.3390/jcm14113620 (PMC12155181; doi:10.3390/jcm14113620)
Supplement: Supplementary file 1 [file jcm-14-03620-s001.zip › Supplmentary Material Document S1_publication bias after including unpublished trials.docx]

**Supplementary Material Document S1: Assessment of publication bias**

This supplementary material provides a detailed description of the sensitivity analysis conduction to assess the potential impact of three unpublished trials ^1,2,3^ on publication bias. Since these trials do not have available data, hypothetical scenarios were created to simulate possible outcomes. The following scenarios were considered:

Best-case scenario: Assuming the unpublished studies favor the intervention.

Worst-case scenario: Assuming the unpublished studies oppose the intervention.

Neutral-case scenario: Assuming no differences between the intervention and control.

**Table S1**: Results of Egger’s test for each hypothetical scenario

| **Scenario** | **t-value** | **df** | **p-value** | **Bias estimate** | **Interpretation** |
| --- | --- | --- | --- | --- | --- |
|  |  |  |  |  |  |
| Original | - 1.14 | 6 | 0.2977 | -1.0176 | No significant evidence of publication bias |
| Best-case scenario | -0.72 | 9 | 0.4873 | -0.5732 | Reduced asymmetry, no significant bias |
| Worst-case scenario | -1.35 | 9 | 0.2121 | -1.2394 | Slightly increased asymmetry, no significant bias |
| Neutral scenario | -1.03 | 9 | 0.3317 | -0.8234 | No substantial change, no significant bias |

1. Effect of thermal softening pretreatment of double-lumen endobronchial tube combined with magnesium sulfate gargle on postoperative sore throat

https://trialsearch.who.int/Trial2.aspx?TrialID=ChiCTR2300072980

1. The effects of thermal softening on postoperative sore throat

https://trialsearch.who.int/Trial2.aspx?TrialID=KCT0002493

1. Effect of warm endotracheal tube insertion on post operation sore throat and hoarseness

https://trialsearch.who.int/Trial2.aspx?TrialID=IRCT201708294365N22
