# Supplementary material for: The Effect of Thermal-Softened Endotracheal Tubes on Postoperative Sore Throat and Other Complications—A Systematic Review and Meta-Analysis
Source: J Clin Med. 2025 May 22;14(11):3620. doi: 10.3390/jcm14113620 (PMC12155181; doi:10.3390/jcm14113620)
Supplement: Supplementary file 1 [file jcm-14-03620-s001.zip › suppplementary Material Table S1_search strategy.docx]

Supplementary Material Table S1. Search strategy for record extraction

Pubmed

|  | Key words or MeSH terms | Results retrieved |
| --- | --- | --- |
| #1 | “thermal soften*”[tw] OR “soften*”[tw] OR “prewarm*”[tw] | 12073 |
| #2 | ("Anesthesia, Endotracheal"[Mesh]) OR "Intubation, Intratracheal"[Mesh] OR “endotracheal tube”[all] OR “endobronchial tube”[all] OR "endotracheal"[All Fields] OR "endotracheally"[All Fields] | 61046 |
| #3 | "pharyngitis"[MeSH Terms] OR "pharyngitis"[All Fields] OR ("sore"[All Fields] AND "throat"[All Fields]) OR "sore throat"[All Fields] OR ("throat"[All Fields] AND "pain"[All Fields] OR "throat pain"[All Fields]) OR "hoarseness"[MeSH Terms] OR "hoarseness"[All Fields] OR "hoarse"[All Fields] OR "hoarsenesses"[All Fields] OR "hoarsness"[All Fields] | 35784 |
| #4 | #1 AND #2 AND #3 AND randomized controlled trial | 9 |

Embase

|  | Key words or Emtree terms | Results retrieved |
| --- | --- | --- |
| #1 | thermal AND soften* OR soften* OR prewarm* | 12406 |
| #2 | 'endotracheal tube'/exp OR 'endotracheal tube' OR 'endotracheal intubation'/exp OR 'endotracheal intubation' OR 'nasotracheal intubation'/exp OR 'nasotracheal intubation' OR 'endobronchial intubation'/exp OR 'endobronchial intubation' OR 'respiratory tract intubation'/exp OR 'respiratory tract intubation' | 105153 |
| #3 | ‘sore throat’/exp OR ‘sore throat’ OR 'hoarseness'/exp OR ‘hoarse’ OR ‘hoarseness’ | 50024 |
| #4 | #1 AND #2 AND #3 AND randomized control trial | 11 |

Cochrane library

|  | Key words or MeSH terms | Results retrieved |
| --- | --- | --- |
| #1 | (thermal soften*):ti,ab,kw OR (soften*):ti,ab,kw OR (prewarm*):ti,ab,kw | 1053 |
| #2 | MeSH descriptor: [Anesthesia, Endotracheal] explode all trees OR MeSH descriptor: [Intubation, Intracheal] explode all trees OR MeSH descriptor [Intubation] explode all trees OR (endotracheal tube):ti,ab,kw OR (“endotracheal”)ti,ab,kw OR (intubation):ti,ab,kw OR (“endobronchial tube”):ti,ab,kw OR (endobronchial):ti,ab,kw | 28539 |
|  |  |  |
| #3 | MeSH descriptor: [Pharyngitis] explode all trees OR (pharyngitis):ti,ab,kw OR (sore throat):ti,ab,kw OR (“sore throat”) OR (throat pain):ti,ab,kw OR MeSH descriptor: [Hoarseness] explode all trees OR (“hoarseness”):ti,ab,kw OR (“hoarse”):ti,ab,kw OR (hoarse*):ti,ab,kw OR (injur*):ti,ab,kw OR MeSH descriptor: [Wounds and injuries] explode all trees | 110027 |
| #4 | #1 AND #2 AND #3 | 29 |
