# Supplementary figures and images for: The Effect of Thermal-Softened Endotracheal Tubes on Postoperative Sore Throat and Other Complications—A Systematic Review and Meta-Analysis
Source: J Clin Med. 2025 May 22;14(11):3620. doi: 10.3390/jcm14113620 (PMC12155181; doi:10.3390/jcm14113620)

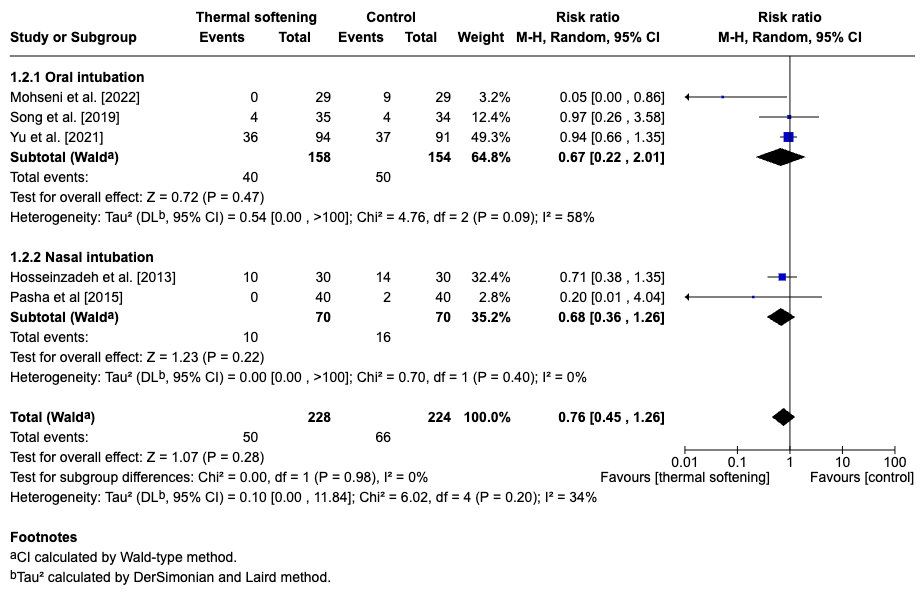

Supplement: Supplementary file 1 [file jcm-14-03620-s001.zip › Supplementary Material Figure S1_sore throat oral vs nasal subgroup analysis.png]
